# Comparison between DOACs and warfarin for left atrial thrombus in atrial fibrillation patients

**DOI:** 10.1016/j.ijcha.2025.101745

**Published:** 2025-07-08

**Authors:** Akihiro Sunaga, Daisaku Nakatani, Katsuki Okada, Hirota Kida, Yuki Matsuoka, Daisuke Sakamoto, Tetsuhisa Kitamura, Nobuaki Tanaka, Yasuyuki Egami, Masaharu Masuda, Tetsuya Watanabe, Hitoshi Minamiguchi, Takafumi Oka, Koichi Inoue, Shungo Hikoso, Yohei Sotomi, Yasushi Sakata

**Affiliations:** a Department of Cardiovascular Medicine, Osaka University Graduate School of Medicine, Osaka, Japan; b Department of Transformative System for Medical Information, Osaka University Graduate School of Medicine, Osaka, Japan; c Department of Social and Environmental Medicine, Osaka University Graduate School of Medicine, Osaka, Japan; d Cardiovascular Center, Sakurabashi Watanabe Hospital, Osaka, Japan; e Division of Cardiology, Osaka Rosai Hospital, Sakai, Japan; f Cardiovascular Center, Kansai Rosai Hospital, Amagasaki, Japan; g Division of Cardiology, Osaka General Medical Center, Osaka, Japan; h Cardiovascular Division, Osaka Police Hospital, Osaka, Japan; i Cardiovascular Division, National Hospital Organization Osaka National Hospital, Osaka, Japan; j Department of Cardiovascular Medicine, Nara Medical University, Japan

**Keywords:** Left atrial thrombus, Atrial fibrillation, Anticoagulation, TEE, LAT resolution

## Abstract

**Background:**

Atrial fibrillation (AF) is a major risk factor for thromboembolic events, with left atrial thrombus (LAT) formation occurring despite oral anticoagulant (OAC) therapy in some patients. Direct oral anticoagulants (DOACs) have demonstrated efficacy in preventing thrombosis; however, their role in LAT resolution compared to warfarin remains unclear.

**Methods:**

This retrospective, multicenter study analyzed 260 AF patients with transesophageal echocardiography (TEE)-confirmed LAT among 17,436 AF patients who underwent TEE. Patients were categorized into DOAC and warfarin groups. LAT resolution, ischemic stroke/systemic embolism, major bleeding, and all-cause death were evaluated. The warfarin group was further stratified by time in therapeutic range (TTR) (<60 % and ≥ 60 %), and the DOAC group by dose (standard and low).

**Results:**

During a median follow-up of 386 [367, 413] days, LAT resolution was achieved in 62 % of patients, significantly higher in the DOAC group (74 % vs. 54 %, P = 0.001). Standard-dose DOACs had the highest resolution rates, while TTR < 60 % had the lowest (79 % vs. 49 %). DOACs were independently associated with higher LAT resolution (OR = 2.91 [1.32–6.38], P = 0.008) and fewer bleeding events (OR = 0.26 [0.08–0.80], P = 0.019).

**Conclusions:**

DOAC therapy was associated with higher LAT resolution rates and showed a fewer bleeding events compared to warfarin. DOACs may serve as first-line therapy for LAT.

## 1 Introduction

Atrial fibrillation (AF) is the most common cardiac arrhythmia and a well-established risk factor for thromboembolic events. [1] The pathophysiology of AF includes blood stasis, a hypercoagulable state, and prothrombotic endothelial changes, [2,3] all of which contribute significantly to the formation of left atrial thrombi (LAT). The left atrial appendage (LAA) is the primary site of thrombus formation in such cases. [[4], [5], [6], [7]] While oral anticoagulant (OAC) therapy has effectively reduced the incidence of LAT, a small proportion of patients—approximately 3 %—continue to develop LAT despite ongoing OAC treatment. [5,[7], [8], [9], [10]].

Direct oral anticoagulants (DOACs) have been widely used for embolism prevention since their introduction due to their efficacy, safety, low risk of drug and food interactions, and the convenience of not requiring monitoring. [[11], [12], [13], [14]] In addition to embolism prevention, DOACs have also been applied in the treatment of conditions such as venous thromboembolism. [[15], [16], [17]] However, the comparison of efficacy between DOACs and warfarin for LAT resolution in patients with LAT, as well as the optimal dosing strategies for DOACs, remains insufficiently studied.

## 2 Method

### 2.1 Study design

This study was a retrospective, multicenter, observational study conducted using registry data of AF patients with LAT detected by transesophageal echocardiography (TEE). The registry was organized by the Osaka Cardiovascular Conference (OCVC) with the participation of six high-volume hospitals comprised by the OCVC-arrhythmia team. The OCVC organization and data collection methods for this registry have been described elsewhere. [[8], [9], [10]].

**Fig. 1:**
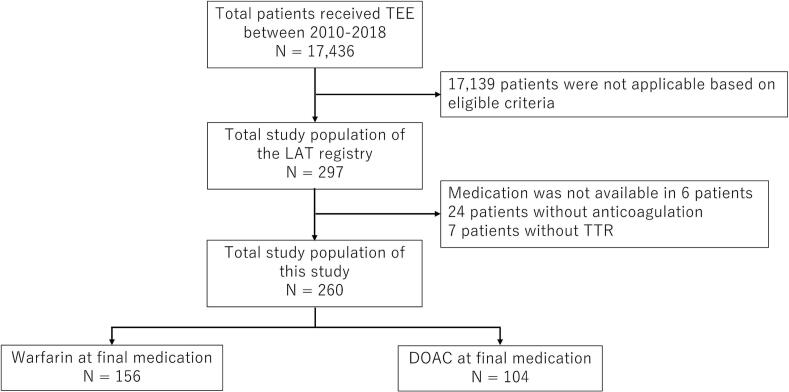
Patient flow chart TEE, transesophageal echocardiography; LAT, left atrial thrombus; TTR, time in therapeutic range; DOAC, direct oral anticoagulation.

In brief, AF patients with TEE-detected LAT between January 2010 and March 2018 were enrolled in the registry, excluding those with a life expectancy of less than 3 months. Among 17,436 AF patients who underwent TEE during this period, 297 (1.7 %) were eligible for inclusion in the registry. Of these, data on final medication were unavailable for 6 patients, 24 patients were not on anticoagulants, and time in therapeutic range (TTR) data were unavailable for 7 patients. These 37 cases were excluded, leaving 260 patients for analysis (Fig. 1).

Final medication was defined as the medication used at the time of LAT resolution in patients with resolved LAT or the medication at the time of the final follow-up in patients without LAT resolution. The 260 patients were divided into two groups based on their final medication: warfarin or DOACs.

### 2.2 Left atrial thrombi

At the time of enrollment, the presence of thrombus was confirmed in all cases using TEE. During follow-up, the presence or resolution of thrombus was assessed using TEE or multi-detector computed tomography (MDCT).

In TEE examinations, LAT was defined as discrete echo-dense masses in the left atrium or left atrial appendage, characterized by echo densities distinct from the adjacent endocardium and independent motion relative to the chamber wall. [18] MDCT was performed using a two-phase scanning protocol with contrast enhancement to improve the specificity of detection. LAT was defined as the presence of residual left atrial appendage filling defects observed in the delayed phase. [19].

### 2.3 Dose of DOACs

The standard doses of DOACs are defined as 300 mg/day for dabigatran, 15 mg/day for rivaroxaban, 10 mg/day for apixaban, and 60 mg/day for edoxaban. The low doses refer to the recommended lower doses based on factors such as renal function, age, and body weight: 220 mg/day for dabigatran, 10 mg/day for rivaroxaban, 5 mg/day for apixaban, and 30 mg/day for edoxaban.

### 2.4 Data collection and follow-up

All data were retrospectively collected from patient medical records. Due to the retrospective nature of the study, written informed consent was not obtained; instead, informed consent was obtained through an opt-out method based on a statement displayed on the institutional website, in compliance with Japanese clinical research guidelines. This study adhered to the ethical principles outlined in the Declaration of Helsinki.

Treatment decisions were made at the discretion of the attending physician in accordance with practical guidelines. [20] Clinical and thrombus follow-up were conducted following each center’s standard of care, with LAT-related data retrospectively retrieved from medical records. Details of anticoagulation therapy were reviewed at enrollment and again at the final follow-up. The follow-up period was set to 1 year after LAT detection.

In patients treated with warfarin, the TTR was calculated as a standard measure of warfarin therapy. TTR incorporates both the frequency of prothrombin time international normalized ratio (PT-INR) measurements and their values to estimate daily PT-INR values and calculates the percentage of time each patient’s PT-INR remained within the therapeutic range. [21] The therapeutic PT-INR range was defined as 2.0–3.0 in this study in accordance with the global standards. [4,6,22].

### 2.5 Endpoints

The endpoints of the study included LAT resolution, ischemic stroke or systemic thromboembolism, major bleeding, and all-cause death. LAT resolution was confirmed using TEE or MDCT. Patients who underwent surgical removal of the LAT were considered as not achieving LAT resolution. Ischemic stroke was defined as atherothrombotic, cardioembolic, or lacunar infarction with a new focal neurological deficit of vascular origin lasting > 24 h. [23] Systemic embolism was defined as acute vascular occlusion of an extremity or organ. Major bleeding was defined according to the International Society of Thrombosis and Hemostasis bleeding criteria [24] and included bleeding events requiring hospitalization.

### 2.6 Statistical analysis

Categorical variables were expressed as counts (percentages) and compared using the chi-squared test or Fisher’s exact test, as appropriate. Continuous variables were reported as mean (standard deviation) or median [interquartile range] and compared using the Student’s *t*-test, Mann-Whitney *U* test, or paired *t*-test, depending on the data distribution. Bonferroni correction was applied for multiple comparisons. Logistic regression analysis was performed to evaluate the impact of DOAC use compared to warfarin use on LAT resolution and major bleeding. The analysis of LAT resolution was adjusted for thrombus formation risk factors [25] and factors associated with successful LAT resolution, [9] including age, female sex, body mass index, heart failure, paroxysmal atrial fibrillation, stroke or transient ischemic attack, diabetes mellitus, prior myocardial infarction, malignancy, left atrial appendage velocity, and creatinine. The analysis of major bleeding was adjusted for bleeding risk factors, [10,25] including age, female sex, hypertension, creatinine, and antiplatelet use. Based on a previous report showing LAT resolution in 60 of 107 warfarin-treated patients and 39 of 50 DOAC-treated patients, [26] the required total sample size to demonstrate the superiority of DOACs over warfarin for LAT resolution was calculated to be 120 patients with a power of 80 % and a significance level of 5 %. Therefore, this study includes a sufficient number of cases to demonstrate statistical significance. Kaplan–Meier analysis was used to compare the outcomes of thromboembolism, major bleeding, and all-cause mortality. Statistical significance was defined as a P-value < 0.05. All statistical analyses were conducted using R software (version 4.4.1; R Foundation for Statistical Computing).

## 3 Result

### 3.1 Baseline characteristics

Among the 260 patients, 104 were on DOACs at the time of final medication, while 156 were on warfarin. The warfarin group was older and had a lower body mass index compared to the DOAC group. Patients in the warfarin group exhibited higher prevalence rates of heart failure, prior stroke or transit ischemic attack, and myocardial infarction, along with higher CHA_2_DS_2_-VASc scores. Additionally, the warfarin group had lower hemoglobin and albumin levels and higher D-dimer and C-reactive protein levels. The left atrial diameter was also larger in the warfarin group. The use of antiplatelet agents, beta-blockers, and diuretics was more common in the warfarin group. There were no significant differences between the two groups regarding sex, hypertension, diabetes mellitus, malignancy, left ventricular ejection fraction, brain natriuretic peptide levels, or the presence of moderate or severe mitral regurgitation (Table 1).

**Table 1:** Baseline patient characteristics.

	Warfarin N = 156	DOAC N = 104	P value
Age, years	71 [64, 78]	67 [61, 75]	0.040
Female, n (%)	50 (32)	28 (27)	0.456
Body mass index, kg/m^2^	23.5 [20.9, 26.2]	24.6 [22.1, 27.6]	0.008
Medical History			
PAF, n (%)	51 (33)	34 (33)	>0.999
Heart failure, n (%)	101 (65)	43 (41)	<0.001
Hypertension, n (%)	104 (67)	71 (68)	0.893
Diabetes mellitus, n (%)	61 (39)	38 (37)	0.774
Stroke or TIA, n (%)	61 (39)	27 (26)	0.039
Prior MI, n (%)	14 (9.0)	2 (1.9)	0.040
Malignancy, n (%)	23 (15)	10 (9.6)	0.296
CHADS_2_ score	2 [1,3]	2 [1,3]	0.014
CHA_2_DS_2_-Vasc score	4 [3,5]	3 [2,4]	0.001
Laboratory data			
Hemoglobin, g/dL	13.6 [11.4, 15.2]	14.3 [12.9, 15.3]	0.030
D-dimer, μg/mL	1.15 [0.52, 3.70]	0.50 [0.21, 1.46]	0.001
BNP, pg/mL	315 [160, 577]	227 [109, 453]	0.061
Creatinine, mg/dL	1.00 [0.81, 1.30]	0.90 [0.72, 1.11]	0.004
Albumin, d/dL	3.80 [3.40, 4.10]	4.00 [3.60, 4.15]	0.009
C-reactive protein, mg/dL	0.34 [0.12, 1.31]	0.20 [0.10, 0.48]	0.021
Transthoracic echocardiography			
LVEF, %	50 [31, 61]	56 [39, 62]	0.059
Left atrial diameter, mm	48 [43, 52]	44 [40, 49]	<0.001
MR ≥ moderate, n (%)	61 (44)	34 (36)	0.270
Transesophageal echocardiography			
Rhythm at examination			0.025
Atrial fibrillation, n (%)	107 (69)	78 (75)	
Sinus, n (%)	9 (5.8)	12 (12)	
Unknown, n (%)	40 (26)	14 (14)	
Left atrial appendage velocity, cm/s	18 [15,24]	21 [15,30]	0.132
Spontaneous echo contrast, n (%)	110 (71)	71 (70)	0.894
Thrombus characteristics			
Maximum length, mm	13.6 [9.7, 20.0]	11.0 [7.8, 17.0]	0.040
Maximum width, mm	10.0 [6.2, 12.6]	8.0 [5.0, 11.0]	0.039
Number of thrombi ≧ 2, n (%)	8 (5.2)	3 (2.9)	0.575
Mobile thrombi, n (%)	23 (15)	24 (23)	0.122
Pedunculated thrombi, n (%)	5 (3.2)	1 (1.0)	0.448
Medication			
Antiplatelet, n (%)	54 (35)	11 (11)	<0.001
ACE-I or ARB, n (%)	65 (42)	44 (42)	>0.999
Ca blocker, n (%)	58 (37)	47 (45)	0.263
Beta blocker, n (%)	90 (58)	46 (44)	0.040
Diuretics, n (%)	91 (59)	44 (42)	0.014
NSAIDs, n (%)	6 (4.1)	3 (2.9)	0.883

DOAC, direct oral anticoagulant; PAF, paroxysmal atrial fibrillation; TIA, transient ischemic attack; MI, myocardial infarction; BNP, brain natriuretic peptide; LVEF, left ventricular ejection fraction; MR, mitral regurgitation; ACE-I, angiotensin converting enzyme inhibitor; ARB, angiotensin II receptor blocker; NSAIDs, non-steroidal anti-inflammatory drugs.

Thrombus size, including both long and short axes, was significantly larger in the warfarin group. However, no differences were observed between the groups in terms of thrombus mobility, morphology, number of thrombi, or left atrial appendage flow velocity (Table 1).

The 156 patients in the warfarin group were further divided into two subgroups based on TTR: 104 patients with TTR ≥ 60 % and 52 patients with TTR < 60 %. Similarly, the 104 patients in the DOAC group were divided into two subgroups: 22 patients receiving low-dose DOACs and 82 patients receiving standard-dose DOACs. A comparison of baseline characteristics revealed significant differences in age, body mass index, heart failure, stroke or transient ischemic attack, malignancy, CHADS_2_ score, CHA_2_DS_2_-VASc score, D-dimer, B-type natriuretic peptide, creatinine, albumin, C-reactive protein, left atrial diameter, the presence of two or more thrombi, and antiplatelet use (Supplementary Table 1). Regarding baseline anticoagulant use, there was no significant difference in the proportion of patients not receiving anticoagulants; however, significant differences were observed for warfarin and DOAC use (Supplementary Table 2).

### 3.2 Oral anticoagulant prescription

The number of patients not receiving anticoagulants at the time of thrombus detection did not differ significantly between the groups (Table 2). However, patients in the warfarin group were significantly more likely to be on warfarin and less likely to be on DOACs at the time of thrombus detection. At the time of final medication, 79 % of patients in the DOAC group were on the standard dose. The median TTR in the warfarin group was 34 %, with 33 % of patients achieving a TTR ≥ 60 %.

**Table 2:** Details of oral anticoagulants.

	Warfarin N = 156	DOAC N = 104	P Value
OAC at baseline			
None, n (%)	26 (17)	15 (14)	0.755
Warfarin, n (%)	116 (74)	21 (20)	<0.001
DOAC, n (%)	14 (9.0)	68 (65)	<0.001
Dabigatran, n (%)	4 (2.6)	15 (14)	0.001
Rivaroxaban, n (%)	3 (1.9)	23 (22)	<0.001
Apixaban, n (%)	5 (3.2)	17 (16)	<0.001
Edoxaban, n (%)	2 (1.3)	13 (13)	<0.001
OAC at final follow-up			
DOAC standard, n (%)	0 (0)	82 (79)	<0.001
DOAC low, n (%)	0 (0)	22 (21)	<0.001
Dabigatran, n (%)	0 (0)	36 (35)	<0.001
Rivaroxaban, n (%)	0 (0)	24 (23)	<0.001
Apixaban, n (%)	0 (0)	39 (38)	<0.001
Edoxaban, n (%)	0 (0)	5 (4.8)	0.021
TTR, %	34 [12, 74]	NA	NA
TTR ≧ 60, n (%)	52 (33)	NA	NA

DOAC, direct oral anticoagulant; OAC, oral anticoagulation; TTR, time in therapeutic range.

### 3.3 Outcomes

**Fig. 2:**
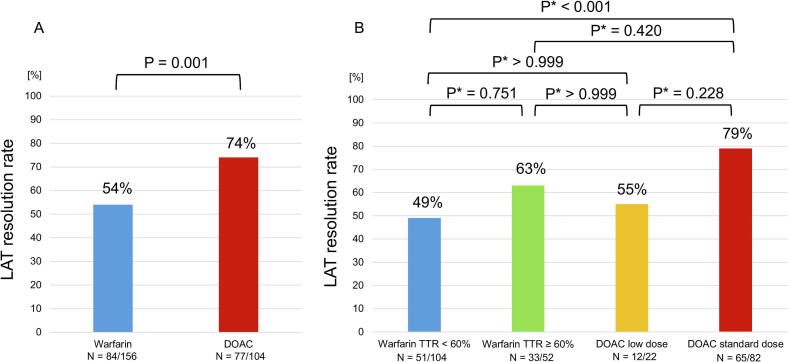
Comparison between DOAC and warfarin for LAT resolution The incidence of LAT resolution during the observation period was compared. A, The red bar graph represents the LAT resolution rate with DOACs, while the blue bar graph represents the LAT resolution rate with warfarin. B, The red bar graph represents the LAT resolution rate with standard-dose DOACs, the yellow bar graph represents the LAT resolution rate with low-dose DOACs, the green bar graph represents the LAT resolution rate with warfarin with TTR ≥ 60 %, and the blue bar graph represents the LAT resolution rate with warfarin with TTR < 60 %. P* indicates the P value adjusted by Bonferroni correction. Statistical significance was defined as a P-value < 0.05. DOAC, direct oral anticoagulant; LAT, left atrial thrombus; TTR, time in therapeutic range. (For interpretation of the references to colour in this figure legend, the reader is referred to the web version of this article.)

**Fig. 3:**
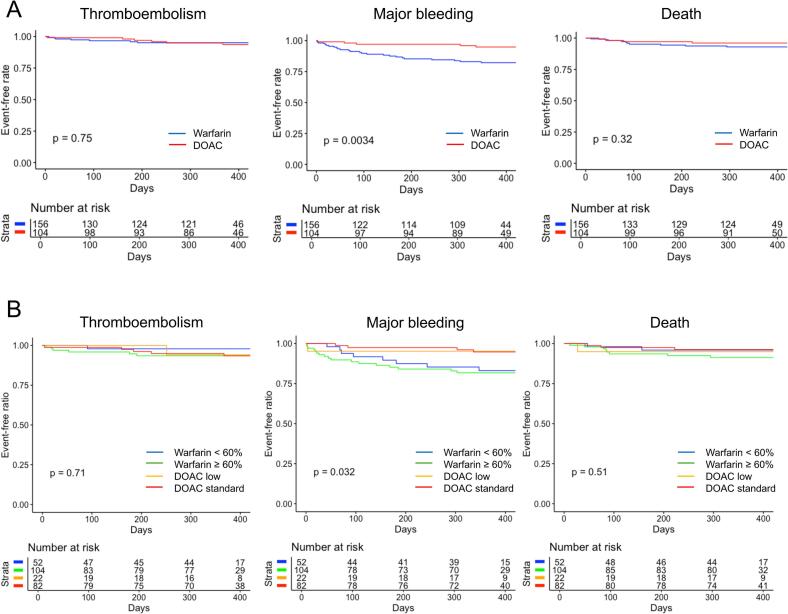
Comparison between DOAC and warfarin for outcomes The outcomes were compared using Kaplan-Meier analysis. A, The red line represents DOAC, while the blue line represents warfarin. B, The red line represents standard-dose DOACs, the yellow line represents low-dose DOACs, the green line represents warfarin with TTR ≥ 60 %, and the blue line represents warfarin with TTR < 60 %.DOAC, direct oral anticoagulant; TTR, time in therapeutic range. (For interpretation of the references to colour in this figure legend, the reader is referred to the web version of this article.)

During a median follow-up of 386 [367, 413] days, LAT resolution was observed in 161 cases (62 %) overall, with significantly higher resolution rates in the DOAC group (Fig. 2A). Kaplan-Meier analysis revealed no significant differences between the groups in terms of all-cause mortality or thromboembolic events; however, bleeding events were significantly less frequent in the DOAC group (Fig. 3A).

Further analysis divided the warfarin group into TTR < 60 % and TTR ≥ 60 % subgroups and the DOAC group into standard-dose and low-dose subgroups. The DOAC standard-dose group showed the highest LAT resolution rate, whereas the TTR < 60 % subgroup had the lowest rate (Fig. 2B). Kaplan-Meier analysis showed no significant differences in thromboembolic events or all-cause mortality, but there was a significant difference in major bleeding events among the groups (Fig. 3B).

We also analyzed the impact of anticoagulant therapy transition patterns on LAT resolution. Patients were categorized into four groups: those who were on warfarin at baseline and remained on warfarin at the end (warfarin to warfarin), those who were on DOACs at baseline and remained on DOACs at the end (DOAC to DOAC), those who switched from warfarin to DOACs, and those who switched from DOACs to warfarin. Multiple comparisons were performed using Bonferroni correction. A significant difference was observed only between the warfarin-to-warfarin group and the DOAC-to-DOAC group, while no significant differences were found between the other groups (Supplementary Figure).

Univariable and multivariable logistic regression analyses demonstrated that DOAC use was significantly associated with higher LAT resolution rates and fewer major bleeding events compared to warfarin (Table 3). Additionally, standard-dose DOAC use was significantly associated with higher LAT resolution rates compared to warfarin with TTR ≥ 60 % in both univariable and multivariable analyses. For major bleeding, standard-dose DOACs were associated with significantly fewer bleeding events in univariable analysis, while multivariable analysis indicated a trend toward fewer bleeding events (Table 4).

**Table 3:** Logistic regression analysis using warfarin as the reference.

	OR [95 %CI] Crude	P	OR [95 %CI] Adjusted	P
LAT resolution				
Warfarin	Ref		Ref	
DOAC	2.44 [1.42–4.19]	0.001	2.91 [1.32–6.38]*	0.008
Major bleeding				
Warfarin	ref		ref	
DOAC	0.26 [0.10–0.72]	0.009	0.26 [0.08–0.80]**	0.019

*Adjusted with age, female, body mass index, heart failure, paroxysmal atrial fibrillation, stroke or transit ischemic attack, diabetes mellitus, prior to myocardial infarction, malignancy, left atrial appendage velocity, and creatinine.

**Adjusted with age, female, hypertension, creatinine, and antiplatelet.

OR, odds ratio; CI, confidence interval; LAT, left atrial thrombus; DOAC, direct oral anticoagulant.

**Table 4:** Logistic regression analysis using warfarin ≥ TTR 60 % as the reference.

	OR [95 %CI] Crude	P	OR [95 %CI] Adjusted	P
LAT resolution				
Warfarin ≥ TTR 60 %	Ref		Ref	
Warfarin < TTR 60 %	0.55 [0.28–1.10]	0.090	0.61 [0.25–1.52]*	0.290
DOAC low dose	0.69 [0.25–1.90]	0.474	0.83 [0.19–3.70]*	0.812
DOAC standard dose	2.20 [1.01–4.79]	0.047	3.04 [1.09–8.50]*	0.034
Major bleeding				
Warfarin ≥ TTR 60 %	Ref		Ref	
Warfarin < TTR 60 %	0.93 [0.37–2.32]	0.877	0.97 [0.38–2.50]**	0.948
DOAC low dose	0.24 [0.03–1.94]	0.182	0.22 [0.02–1.91]**	0.168
DOAC standard dose	0.26 [0.08–0.81]	0.020	0.27 [0.06–1.11]**	0.069

*Adjusted with age, female, body mass index, heart failure, paroxysmal atrial fibrillation, stroke or transit ischemic attack, diabetes mellitus, prior to myocardial infarction, malignancy, left atrial appendage velocity, and creatinine.

**Adjusted with age, female, hypertension, creatinine, and antiplatelet.

OR, odds ratio; CI, confidence interval; LAT, left atrial thrombus; DOAC, direct oral anticoagulant; TTR, time in therapeutic range.

## 4 Discussion

### 4.1 Main findings

Among 17,436 AF patients who underwent TEE, 1.7 % were found to have LAT. Anticoagulant therapy achieved LAT resolution in 161 cases (62 %). Patients treated with DOACs for LAT showed higher LAT resolution rates and lower bleeding rates compared to those treated with warfarin. Furthermore, patients on standard-dose DOACs had significantly higher LAT resolution rates and a tendency for fewer bleeding events compared to patients maintaining a TTR ≥ 60 % with warfarin.

This study is significant as it leverages the world’s largest registry of LAT patients, including 297 cases out of 17,436 AF patients who underwent TEE, surpassing a *meta*-analysis comprising 14,653 cases from 35 studies. [5].

### 4.2 Prevalence of left atrial thrombi

The prevalence of LAT in this study was 1.7 %, which is slightly lower than previously reported [5] In a study conducted by our group involving 3,139 TEE patients from 2010 to 2012, the prevalence of left atrial thrombus was 3.2 %. [[8], [9], [10]] That earlier study reflected a period when warfarin was almost exclusively used, whereas the current study spans a period during which DOACs became available. This suggests that the use of DOACs might have contributed to reducing the risk of developing LAT. As data on patients without LAT were not collected, the reasons for the low prevalence of LAT cannot be statistically explained.

### 4.3 Doacs versus warfarin for LAT treatment

Many studies report LAT resolution rates of 60–80 % in LAT patients, with no significant differences between DOACs and warfarin. [[27], [28], [29]] However, these studies are all smaller in scale compared to the present study. With a relatively large sample size, this study demonstrated that DOACs were associated with higher LAT resolution rates and fewer bleeding events compared to warfarin. The LAT resolution rate was generally consistent with previously reports.

A *meta*-analysis integrating small-scale studies comparing thrombus resolution rates between DOACs and warfarin demonstrated the superiority of DOACs, [30] and our study yielded consistent findings. Notably, our study included the largest number of patients among those studies, thereby providing more robust evidence supporting the efficacy of DOACs.

The low median TTR of 34 % likely contributed to the lower LAT resolution rate in the warfarin group and the higher rate in the DOAC group. Possible explanations are as follows: 1) Patients with inherently unstable PT-INR control, who are more prone to thrombosis, were selectively included, resulting in a selection bias; 2) Because this was a retrospective analysis, it may just reflect a real-world scenario; and 3) Some physicians might have tried to target a PT-INR > 3 in order to effectively resolve the LAT. Such cases would fall outside the therapeutic range, potentially contributing to the low TTR observed.

Multivariable analysis revealed that patients on standard-dose DOACs had a higher likelihood of achieving LAT resolution. Compared to warfarin with TTR ≥ 60 %, standard-dose DOACs were associated with higher LAT resolution rates and a trend toward fewer bleeding events.

In the treatment of venous thromboembolism, drugs like apixaban and rivaroxaban are administered only at standard doses, [15,16] as there are no dose reduction criteria like those used for thrombosis prevention in AF patients. It is conceivable that the appropriate dose may differ between thrombosis prevention in patients without thrombus and treatment in patients with an existing thrombus. For patients with LAT, the optimal DOAC dosage may align with that used for venous thromboembolism treatment; however, this hypothesis would require validation through prospective studies.

Based on the findings of this study, if standard-dose DOACs can be administered, they should be prioritized. If standard-dose DOACs cannot be used, warfarin treatment may be considered, provided that stable PT-INR control can be ensured.

### 4.4 Clinical Implication

When left atrial thrombus is detected, standard-dose DOAC therapy may be recommended to achieve better LAT resolution with fewer bleeding events. If standard-dose DOACs cannot be used, warfarin with a TTR ≥ 60 % should be considered.

### 4.5 Limitations

This study includes a large number of cases but is retrospective in nature, and therefore information bias and missing data bias cannot be completely eliminated. TEE was not performed on all atrial fibrillation patients. In real-world clinical settings, it was primarily conducted for thrombus evaluation prior to AF ablation or as part of preoperative assessments for cardiac surgery. Therefore, selection bias is also present. Most cases involved patients who underwent TEE prior to treatments such as AF ablation, excluding those who declined treatment or were too critically ill to undergo the procedure. While TEE is considered the gold standard for detecting thrombi, the possibility of false positives cannot be ruled out.

## 5 Conclusion

DOAC therapy, particularly at standard doses, was associated with higher LAT resolution rates and fewer bleeding events compared to warfarin. DOACs may potentially serve as the first-line therapy for patients with LAT

## CRediT authorship contribution statement

**Akihiro Sunaga:** Writing – review & editing, Writing – original draft, Methodology, Investigation, Formal analysis, Conceptualization. **Daisaku Nakatani:** Supervision. **Katsuki Okada:** Supervision. **Hirota Kida:** Supervision. **Yuki Matsuoka:** Supervision. **Daisuke Sakamoto:** Supervision. **Tetsuhisa Kitamura:** Supervision. **Nobuaki Tanaka:** Supervision. **Yasuyuki Egami:** Supervision. **Masaharu Masuda:** Supervision, Resources, Data curation. **Tetsuya Watanabe:** Supervision. **Hitoshi Minamiguchi:** Supervision. **Takafumi Oka:** Supervision, Resources, Data curation. **Koichi Inoue:** Supervision, Resources, Project administration, Methodology, Investigation, Data curation, Conceptualization. **Shungo Hikoso:** Supervision, Resources, Project administration, Methodology, Investigation, Data curation, Conceptualization. **Yohei Sotomi:** Writing – review & editing, Supervision, Project administration, Conceptualization. **Yasushi Sakata:** Supervision, Resources, Project administration, Methodology, Investigation, Data curation, Conceptualization.

## Funding

None.

## Declaration of competing interest

The authors declare that they have no known competing financial interests or personal relationships that could have appeared to influence the work reported in this paper.
