# Supplementary material for: Comparison between DOACs and warfarin for left atrial thrombus in atrial fibrillation patients
Source: Int J Cardiol Heart Vasc. 2025 Jul 8;59:101745. doi: 10.1016/j.ijcha.2025.101745 (PMC12274838; doi:10.1016/j.ijcha.2025.101745)
Supplement: Supplementary Data 1 [file mmc1.docx]

**Supplementary Table 1: Baseline patient characteristics**

|  | Warfarin  TTR < 60%  N = 104 | Warfarin  TTR ≥ 60%  N = 52 | DOAC  low dose  N = 22 | DOAC  standard dose  N = 82 | P value |
| --- | --- | --- | --- | --- | --- |
| Age, years | 71 [63, 79] | 71 [65, 76] | 76 [71, 81] | 65 [61, 73] | <0.001 |
| Female, n (%) | 33 (32) | 17 (33) | 9 (41) | 19 (2) | 0.335 |
| Body mass index, kg/m^2^ | 23.4 [20.1, 25.5] | 24.3 [21.4, 27.2] | 22.0 [19.8, 24.8] | 25.0 [23.3, 28.4] | <0.001 |
| **Medical History** |  |  |  |  |  |
| PAF, n (%) | 39 (38) | 12 (23) | 9 (41) | 25 (31) | >0.999 |
| Heart failure, n (%) | 70 (67) | 31 (60) | 8 (36) | 35 (43) | 0.002 |
| Hypertension, n (%) | 71 (68) | 33 (64) | 16 (73) | 55 (67) | 0.876 |
| Diabetes mellitus, n (%) | 44 (42) | 17 (33) | 5 (23) | 33 (40) | 0.285 |
| Stroke or TIA, n (%) | 38 (37) | 23 (44) | 9 (41) | 18 (22) | 0.037 |
| Prior MI, n (%) | 11 (11) | 3 (5.8) | 1 (4.5) | 1 (1.2) | 0.069 |
| Malignancy, n (%) | 17 (17) | 6 (12) | 6 (27) | 4 (4.9) | 0.018 |
| CHADS_2_ score | 2 [2, 3] | 2 [1, 3] | 3 [2, 3] | 2 [1, 3.] | 0.018 |
| CHA_2_DS_2_-Vasc score | 4 [3, 5] | 4 [2, 5] | 4 [3, 5] | 3 [2, 4] | <0.001 |
| **Laboratory data** |  |  |  |  |  |
| Hemoglobin, g/dL | 13.6 [11.4, 15.1] | 13.5 [11.2, 15.2] | 14.1 [12.5, 15.1] | 14.3 [12.9, 15.3] | 0.172 |
| D-dimer, μg/mL | 1.21 [0.51, 2.97] | 1.02 [0.57, 4.14] | 0.90 [0.46, 1.48] | 0.50 [0.20, 1.09] | 0.005 |
| BNP, pg/mL | 385 [195, 676] | 255 [92, 383] | 198 [100, 489] | 245 [115, 437] | 0.022 |
| Creatinine, mg/dL | 1.00 [0.81, 1.36] | 0.94 [0.81, 1.20] | 0.80 [0.60, 0.98] | 0.92 [0.77, 1.15] | 0.005 |
| Albumin, d/dL | 3.70 [3.30, 4.05] | 3.95 [3.60, 4.12] | 4.10 [3.60, 4.10] | 4.00 [3.62, 4.18] | 0.007 |
| C-reactive protein, mg/dL | 0.49 [0.16, 1.91] | 0.17 [0.07, 0.39] | 0.18 [0.08, 0.34] | 0.20 [0.10, 0.52] | <0.001 |
| **Transthoracic echocardiography** |  |  |  |  |  |
| LVEF, % | 49 [30, 59] | 56 [35, 63] | 57 [42, 62] | 56 [37, 62] | 0.094 |
| Left atrial diameter, mm | 48 [43, 51] | 50 [45, 56] | 40 [36, 44] | 45 [42, 49] | <0.001 |
| MR ≥ moderate, n (%) | 40 (42) | 21 (49) | 10 (48) | 24 (32) | 0.292 |
| **Transesophageal echocardiography** |  |  |  |  |  |
| Rhythm at examination |  |  |  |  | 0.116 |
| Atrial fibrillation, n (%) | 67 (64) | 40 (77) | 16 (73) | 62 (76) |  |
| Sinus, n (%) | 7 (6.7) | 2 (3.8) | 3 (14) | 9 (11) |  |
| Unknown, n (%) | 30 (29) | 10 (19) | 3 (14) | 11 (13) |  |
| Left atrial appendage velocity, cm/s | 17 [15, 21] | 20 [16, 28] | 19 [12, 26] | 21 [15, 30] | 0.068 |
| Spontaneous echo contrast, n (%) | 68 (65) | 42 (81) | 12 (57) | 59 (72) | 0.126 |
| Thrombus characteristics |  |  |  |  |  |
| Maximum length, mm | 14.0 [9.2, 18.8] | 12.8 [9.8, 23.5] | 8.9 [5.0, 13.0] | 11.3 [8.0, 17.0] | 0.101 |
| Maximum width, mm | 10.0 [6.0, 12.1] | 9.0 [7.0, 13.0] | 6.2 [3.7, 8.9] | 8.3 [5.3, 11.0] | 0.137 |
| Number of thrombi ≧ 2, n (%) | 2 (1.9) | 6 (11.5) | 1 (4.5) | 2 (2.5) | 0.033 |
| Mobile thrombi, n (%) | 15 (14) | 8 (15) | 8 (36) | 16 (20) | 0.099 |
| Pedunculated thrombi, n (%) | 4 (3.8) | 1 (1.9) | 0 (0.0) | 1 (1.2) | 0.557 |
| **Medication** |  |  |  |  |  |
| Antiplatelet, n (%) | 39 (38) | 15 (29) | 4 (18) | 7 (9) | <0.001 |
| ACE-I or ARB, n (%) | 44 (42) | 21 (40) | 9 (41) | 35 (43) | 0.994 |
| Ca blocker, n (%) | 41 (40) | 17 (33) | 11 (50) | 36 (44) | 0.466 |
| Beta blocker, n (%) | 59 (57) | 31 (60) | 9 (41) | 37 (45) | 0.174 |
| Diuretics, n (%) | 61 (59) | 30 (58) | 8 (36) | 36 (44) | 0.068 |
| NSAIDs, n (%) | 28 (29) | 13 (26) | 4 (18) | 27 (33) | 0.554 |

TTR, time in therapeutic range; DOAC, direct oral anticoagulant; PAF, paroxysmal atrial fibrillation; TIA, transient ischemic attack; MI, myocardial infarction; BNP, brain natriuretic peptide; LVEF, left ventricular ejection fraction; MR, mitral regurgitation; ACE-I, angiotensin converting enzyme inhibitor; ARB, angiotensin II receptor blocker; NSAIDs, non-steroidal anti-inflammatory drugs.

**Supplementary Table 2: Details of oral anticoagulants**

|  | Warfarin  TTR < 60%  N = 104 | Warfarin  TTR ≥ 60%  N = 52 | DOAC  low dose  N = 22 | DOAC  standard dose  N = 82 | P Value |
| --- | --- | --- | --- | --- | --- |
| OAC at baseline |  |  |  |  |  |
| None, n (%) | 21 (20) | 5 (9.6) | 4 (18) | 11 (13) | 0.327 |
| Warfarin, n (%) | 76 (73) | 40 (77) | 4 (18) | 17 (21) | <0.001 |
| DOAC, n (%) | 7 (6.7) | 7 (14) | 14 (64) | 54 (66) | <0.001 |
| Dabigatran, n (%) | 1 (1.0) | 3 (5.8) | 5 (23) | 10 (12) | 0.001 |
| Rivaroxaban, n (%) | 3 (2.9) | 0 (0.0) | 1 (4.5) | 22 (27) | <0.001 |
| Apixaban, n (%) | 2 (1.9) | 3 (5.8) | 5 (23) | 12 (15) | 0.001 |
| Edoxaban, n (%) | 1 (1.0) | 1 (1.9) | 3 (14) | 10 (12) | 0.002 |
| OAC at final follow-up |  |  |  |  |  |
| Dabigatran, n (%) | 0 (0.0) | 0 (0.0) | 11 (50) | 25 (31) | <0.001 |
| Rivaroxaban, n (%) | 0 (0.0) | 0 (0.0) | 3 (14) | 21 (26) | <0.001 |
| Apixaban, n (%) | 0 (0.0) | 0 (0.0) | 6 (27) | 33 (40) | <0.001 |
| Edoxaban, n (%) | 0 (0.0) | 0 (0.0) | 2 (9.1) | 3 (3.7) | 0.016 |
| Warfarin, n (%) | 104 (100) | 52 (100.0) | 0 (0.0) | 0 (0.0) | <0.001 |
| TTR ≥ 60, n (%) | 0 (0.0) | 52 (100) | NA | NA | NA |

DOAC, direct oral anticoagulant; OAC, oral anticoagulation; TTR, time in therapeutic range.

Supplementary Figure: Comparison of LAT resolution according to OAC transition

The incidence of LAT resolution during the observation period was compared. Patients were categorized into four groups: those who were on warfarin at baseline and remained on warfarin at final follow-up, those who were on DOACs at baseline and remained on DOACs at final follow-up, those who switched from warfarin to DOACs, and those who switched from DOACs to warfarin. P* indicates the P value adjusted by Bonferroni correction. Statistical significance was defined as a P-value < 0.05.

LAT, left atrial thrombus; OAC, oral anticoagulant; DOAC, direct oral anticoagulant.
